# Verticillium Wilt of Cotton: Identification and Detection of the Causal Pathogen and Its Control

**DOI:** 10.3390/plants15020239

**Published:** 2026-01-13

**Authors:** Duy P. Le, Carlos Trapero, Chi P. T. Nguyen, Thao T. Tran, Donald Gardiner, Andrew Chen

**Affiliations:** 1 New South Wales Department of Primary Industries and Regional Development, Narrabri, NSW 2390, Australia; chinguyen2009@gmail.com; 2 Faculty of Agriculture and Environment Resources, Dong Thap University, Dong Thap 81118, Vietnam; 3 Agronomy Department, University of Cordoba (UCO), 14005 Cordoba, Spain; carlostrapero@uco.es; 4 Faculty of Engineering and Technology, Dong Thap University, Dong Thap 81118, Vietnam; tranthao2009@gmail.com; 5 Queensland Alliance for Agriculture and Food Innovation, The University of Queensland, St Lucia, QLD 4072, Australia; donald.gardiner@uq.edu.au; 6 School of Agriculture and Food Sustainability, The University of Queensland, St Lucia, QLD 4072, Australia; a.chen2@uq.edu.au

**Keywords:** *Gossypium hirsutum*, wilt diseases, vascular discoloration, genomics, integrated management, cotton breeding

## Abstract

*Verticillium wilt* (VW) of cotton caused by the soilborne pathogen *Verticillium dahliae* is a major disease across cotton production worldwide. The disease can result in yield reductions up to 80% on some occasions. *V. dahliae* is an asexual fungus and belongs to a relatively small *Verticillium* genus in the Ascomycota, though both of the mating type idiomorphs are present within some populations. The diversity of *V. dahliae* is widely associated with vegetative compatibility groups (VCGs), of which six different VCGs are recognised. Of these, isolates belonging to VCGs 1, 2, and 4 are globally distributed and associated with a broad host range, including cotton. Approximately 400 plant species have been recorded as hosts of *V. dahliae*. The pathogenicity and virulence of *V. dahliae* in many cases are correlated with VCG designations and hosts of origin. Disease management of VW of cotton still relies on accurate, rapid detection and quantification of *V. dahliae* using both conventional and molecular approaches. The use of resistant cultivars is the most effective and economical control strategy; however, no cultivars confer complete resistance to the disease. Control strategies including cultural, biological, chemical, and induced-resistance approaches have indicated certain degrees of success in minimising disease damage and diminishing the build-up of pathogen inoculum. In this review, we discuss insights into the VW disease of cotton, and the associated pathogen and current control approaches, as well as future research perspectives.

## 1. Introduction

Cotton is an economically important fibre crop belonging to the *Gossypium* genus. There are about 50 species within the *Gossypium* which can be found in a wide range of ecological niches from arid to semi-arid areas of the tropical and subtropical zones [1]. Due to superior fibre quality and quantity traits, upland cotton (*G. hirsutum*) and Pima cotton (*G. barbadense*) are of the dominant cultivated species worldwide [1]. Cotton contributes approximately 40% of the world’s natural fibre [2], with upland cotton providing 95% of lint production [1,3].

Cotton is prone to infection with an array of pathogens and parasites, including fungi, bacteria, viruses, phytoplasmas, and nematodes causing more than 40 known diseases [4]. Of these, a vascular disease known as Verticillium wilt (VW) caused by *Verticillium dahliae* is the most devastating disease in most production countries, including Australia [5], China [6], Turkey [7], and the United States [8]. Occasionally, *V. albo-atrum* was also isolated from diseased cotton plants [9]. *Verticillium dahliae* is a soilborne pathogen in a small genus of ascomycete [10]; however, seedborne transmission of the pathogen is also a potential source of pathogen dissemination [11]. During 2016–2019, the highest average incidence of VW in Queensland (Qld) and New South Wales (NSW), Australia, was 4% and 30%, respectively [12]. In China, the losses of lint cotton yield may be as high as 80% [13]. In Turkey, VW significantly reduced cotton yield by 15.93% [14]. Cotton yield loss due to the VW disease was estimated to be up to 480 million bales over the period of 1990–2014 in the USA [15]. The disease incidence and severity were highly correlated to cultivar genotypes [16,17,18], seeding rates [19], irrigation regimes [20], fertilisation [20,21], soil types [8], and pathogen load [22,23].

Our review aims to provide an overview of significances of the VW disease to cotton production and its population diversity for a better understanding of the causal pathogen. We also summarise effective and reliable approaches for the detection and identification of the pathogen. Finally, we discuss current and future perspectives of control strategies, especially focusing on breeding for resistance.

## 2. Verticillium Wilt Disease

### 2.1. Symptoms

**Figure 1:**
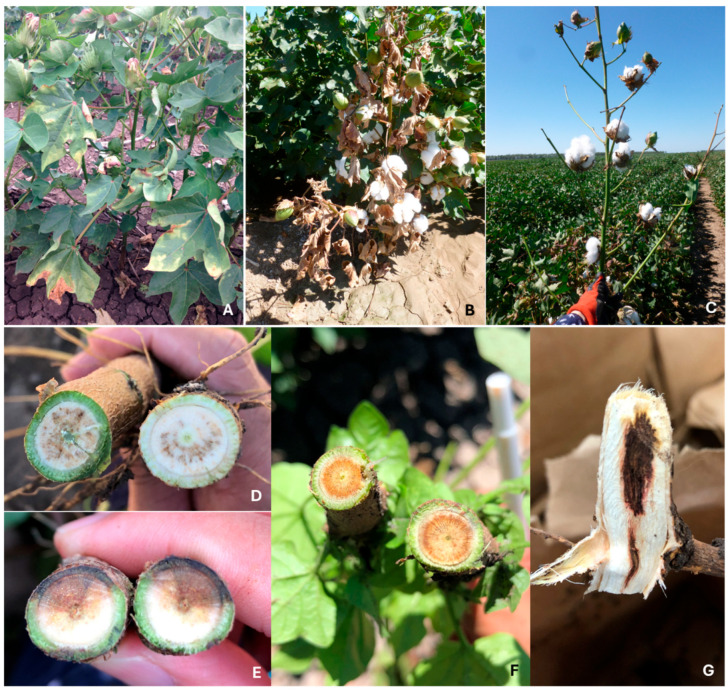
Diagnostic field symptoms to differentiate VW from other diseases in Australian cotton fields. (**A**), foliar symptoms with interveinal chlorosis and marginal necrosis; (**B**), a dead *V. dahliae*-infected plant; (**C**), a completely defoliated cotton plant induced by infection of a *V. dahliae* defoliating pathotype; (**D**), a cross-section showing a typical peppery grey vascular discolouration of a *V. dahliae*-infected plant; (**E**), reddish grey discoloration of vascular tissue occurring in wedges that is characteristic of *Eutypella* infection; (**F**), profound brown discoloration of vascular tissue associated with *Fusarium oxysporum* f. sp. *vasinfectum* infection; (**G**), black necrosis of vascular tissue that is often restricted at the crown, caused by *Berkeleyomyces rouxiae* infection; adopted from Le et al. [27].

Cotton infected with *V. dahliae* often results in characteristic symptoms that allow for the identification of the disease in the field. However, VW symptoms may vary depending on developmental stages, genotypes, plant density, pathogen virulence and pathogen load, and environmental conditions [24]. Symptoms such as wilting and yellowing with necrosis of leaf margins often appear first on lower leaves and then progress upwards and to the whole plants (Figure 1A). Early infected plants remain stunted or killed (Figure 1B). In the field, those symptoms could be misdiagnosed with Fusarium wilt caused by *Fusarium oxysporum* f. sp. *vasinfectum* (Fov) [25]. However, *V. dahliae* infection in cotton often incites grey peppery discoloration in vascular tissue (Figure 1D) that is different from the vascular symptom induced by Fusarium wilt, which is often visualised with a profound brown colour (Figure 1F). Vascular discoloration is of the most diagnostic characteristics to differentiate these two wilt diseases, with a recently characterised wilt disease associated with novel *Eutypella* spp. *Eutypella* spp. infection results in reddish grey discoloration occurring in wedges on lower stems (Figure 1E) [26]. On fewer occasions, the black root rot pathogen *Berkeleyomyces rouxiae* can also induce black vascular discoloration restricted to the crown region (Figure 1G) that can be misdiagnosed as other wilt diseases, including VW [27]. Other symptoms, including part to complete defoliation (Figure 1C) and terminal branch die-back are also often observed in cotton plants infected with either *V. dahliae* or Fov [28]. Due to the overlapping of wilt disease symptoms in the field, pathogen isolation from infected tissue is one of the most reliable diagnostic methods [29].

### 2.2. Economic Impacts

VW is a destructive disease and causes significant losses in cotton worldwide [15,30,31], since 95% of commercial cotton is derived from Upland cotton, which is more susceptible to *V. dahliae* infection than its relative Pima cotton [1]. In Australia, yield losses were not significant in a hot dry 1984/85 season, but losses up to 62% were estimated on some occasions in 2015/16 season [32]. According to CSD [33], VW caused estimated losses in NSW from AUD 1.9 to AUD 3.8 M per season from 2006 to 2010. In the latest survey of Australian cotton consultants, Barker and Coggan [34] revealed that 14% growers were severely affected by VW. In China, Wei et al. [22] reported up to 50% of production areas were affected by VW, resulting in an estimated economic loss of USD 250–310 M annually. In some regions, the loss of lint cotton yield may be as high as 80% [13]. In Alabama and Tennessee, USA, yield losses were also substantial, exceeding 29,100 bales across three seasons from 2014 to 2016 [15]. These production losses resulted in an estimated USD 11.2 M loss of income due to VW. In Turkey, VW also reduced cotton yield by 15.93%, and reduced fibre length and strength [14].

### 2.3. Ecological Impacts

*Verticillium dahliae* is a soilborne pathogen that produces multicellular, melanised resting structures known as microsclerotia inside senescing host plants [35]. Microsclerotia can resist radiation and mycostasis, and remain dormant in soil or plant debris for up to 14 years without the presence of a host plant [35,36,37]. Additionally, *V. dahliae* has a wide host range, infecting more than 400 plant species, including many weed species that can provide a bridge between cropping seasons [10,38,39,40]. Therefore, it appears that once introduced, *V. dahliae* could persist indefinitely in soil due to its tough resting microsclerotia [35,36,37] and wide host range nature [10].

## 3. Verticillium Wilt Pathogens

### 3.1. Taxonomy and Identification

**Figure 2:**
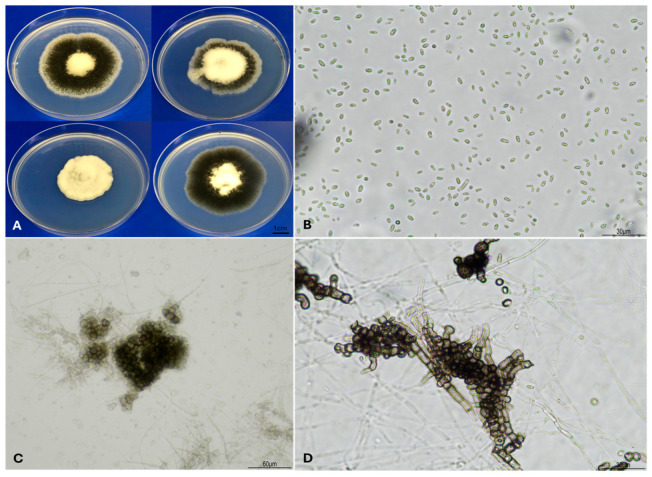
Morphological characteristics of *Verticillium dahliae* recovered from Australian cotton. (**A**), diverse colonial appearances of *V. dahliae* on potato dextrose agar (PDA) do not necessarily correlate to its pathotype designations; (**B**), typical cylindrical, hyaline, smooth-walled conidia produced on PDA; (**C**,**D**), round and elongate microsclerotia observed when *V. dahliae* from Australian cotton was grown on PDA.

VW of cotton is primarily associated with *V. dahliae* [41]; occasionally, *V. albo-atrum* was also isolated from diseased cotton plants [9]. *Verticillium dahliae* belongs to a relatively small genus, *Verticillium*, which encompasses ten species [10]. The genus was first erected in 1816 [42] and approximately 190 species have been assigned to the genus since then [43]. However, a significant number of distant related species have been removed from the genus based on systematic examinations of molecular sequences [43]. Morphologically, *V. dahliae* distinguishes itself from *V. albo-atrum* and other species by producing its characteristic short conidia and round microsclerotia as the only resting structure (Figure 2) [10]. Currently, *V. dahliae* is clustered in the flavnonexudans group, which does not produce yellow hyphal pigments, while *V. albo-atrum* is a member of the flavexudans group [10]. Following phylogenetic analyses, *V. dahliae* and *V. albo-atrum* are clustered in two well-defined groups and both species are now universally recognised as two separate taxa [10,44].

### 3.2. Population Biology

*Verticillium dahliae* belongs to the Ascomycota. However, no evidence of sexual recombination has been recorded in this species [45,46,47]. Consequently, the population structure of *V. dahliae* appeared highly clonal [48,49,50]. Prior to the widespread adoption of molecular markers, the population biology of *V. dahliae* was commonly studied on the basis of VCGs [51,52]. Here, we discuss *V. dahliae* populations recovered from cotton based on VCGs and mating types.

#### 3.2.1. VC Groups

Isolates of *V. dahliae* recovered from various hosts and geographic locations have been assigned to six different VCGs, namely VCG 1 to VCG 6 [53]. VCGs 1, 2, and 4 were subdivided into subgroups A and B based on vigour of their compatibility reactions [54]. These VCGs are globally distributed and associated with a wide host range [29,48,49,55], whereas isolates assigned to VCGs 3, 5, and 6 are less commonly encountered [53,54]. VW of cotton worldwide was attributed to isolates of *V. dahliae* belonging to VCGs 1, 2, and 4 [6,56,57]. In Australia, VCG 1A, VCG 2A, and VCG 4B were recorded [56]. Similar VCG profiles were also reported on cotton grown in China, Greece, Spain, and the USA [6,57,58]. Collado-Romero et al. [51] were the first to identify VCG 1B isolates from cotton grown in Greece; however, the virulence of these isolates on cotton was not assessed. Uncommonly encountered VCG 3 isolates, which were originally obtained from potato in the USA [54], were subsequently reported for the first time on cotton in China [59]. Cotton grown in Israel is known to be infected by isolates within VCGs 2A, 2B, and 4, which were assigned to the non-defoliating pathotype [57,60]. A defoliating pathotype, VCG 1, was identified from Israeli cotton [61]. Similarly, VW of cotton in Turkey was induced by isolates belonging to VCGs 1A, 2A, 2B, and 4B, of which VCG 1A and VCG 2B are more prevalent [62,63]. Discoveries of new VCGs in some cotton-growing regions raised concerns as to whether the pathogen has been introduced recently or if the population diversity was underestimated. It is acknowledged that VCG analyses are laborious and time consuming. Therefore, VCG studies are often limited to a sample range, which limits any comprehensive conclusions from being drawn on the pathogen population [52]. A number of molecular approaches such as amplified fragment length polymorphism (AFLP), microsatellite, multi-gene sequencing, and genotyping-by-sequencing were employed to attempt to infer the relationship between the VCGs and their genetic diversity [49,51,64,65]. However, intraspecific variations were consistently observed among isolates from both within and between VCGs [49,51,64]. Recently, genome-wide data of *V. dahliae* have become available, which will subsequently allow for elucidating a complex relationship between VCGs in *V. dahliae* and genetic evolution [66,67,68,69]. Indeed, genome-wide markers largely support the clonality of the finer VCG groupings but demonstrate that the VCG subgroupings are not indicative of relatedness [70]. To illustrate this, for example, VCG2A is not closely related to VCG2B; VCG2A is in fact more closely related to VCG4B. Moreover, molecular data strongly suggested recombination has occurred within and between lineages [70].

#### 3.2.2. Mating Types

Currently, *V. dahliae* is known for its strictly asexual reproduction, so understanding how genetic diversity is generated within *V. dahliae* remains unclear [71], although recombination is evident [70]. In *V. dahliae*, genes of both mating-type (MAT) idiomorphs have been identified [45,46,70]. Generally, the ratio of two opposite MAT genes is commonly found to be 1:1 in sexually reproducing populations of fungi [46,70]. However, *V. dahliae* populations predominantly harbour the MAT1-2 idiomorph [45,46,47,65,70,72,73]. To a much lesser extent, the MAT1-1 idiomorph was also detected in lettuce–*V. dahliae* isolates [46,72,74]. Although *V. dahliae* isolates carrying the MAT1-2 gene were overwhelmingly reported in most crops, *V. dahliae* populations carrying both MAT1-1 and MAT1-2 were also detected in the same ecological niches [45,46,72,74]. Therefore, sexual recombination within *V. dahliae* populations could occur in nature. Nonetheless, Usami et al. [46] failed to pair up isolates with MAT1-1 and MAT1-2 for sexual reproduction in vitro. Similarly, the clonal *V. dahliae* population was reported, despite the recombination events observed through SNP genotyping analyses of 141 *V. dahliae* isolates from diverse hosts and geographic origins [70]. Consequently, Milgroom et al. [70] concluded that sexual recombination in *V. dahliae* may not occur frequently. Regardless of the presence of both MAT idiomorphs in *V. dahliae*, sexual reproduction has not been detected, so research to elucidate if *V. dahliae* has lost its sexuality or not remains to be addressed. Additionally, as most *V. dahliae* collections have been recovered from diseased plants, it has been suggested that the high abundance of MAT1-2 isolates may be linked to their virulence, which also warrants further research [45,75].

#### 3.2.3. Pathogenicity

**Figure 3:**
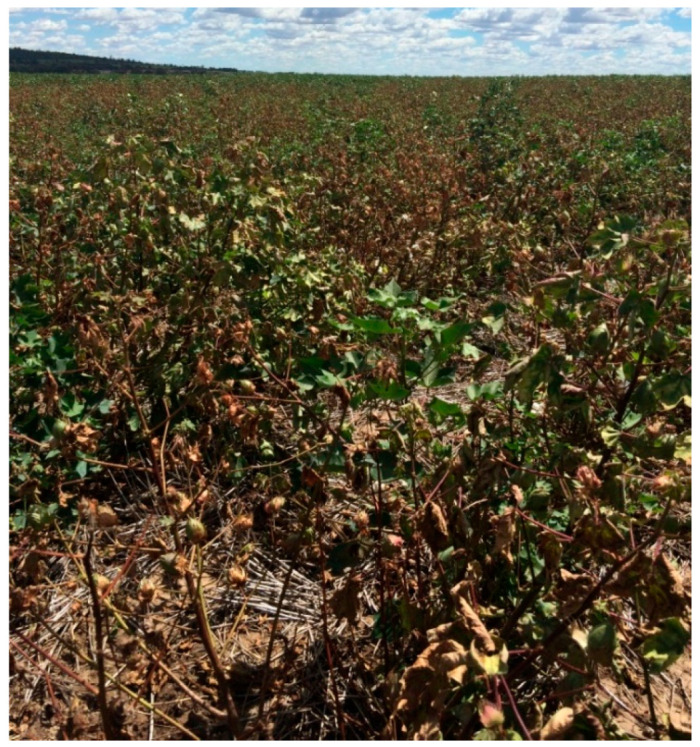
A cotton field with severe symptoms of Verticillium wilt in NSW, Australia, induced by the non-defoliating pathotype.

*Verticillium dahliae* has been reported to infect up to 400 host plant species [76]. In cotton, the virulence of *V. dahliae* was commonly found to be associated with VCGs [7,61,77,78]. Isolates belonging to VCG 1A and VCG 2B were more virulent than isolates assigned to VCG 2A and VCG 4B. VCG 1A and 2B induced severe chlorotic to necrotic foliar symptoms and eventually defoliation and death, whilst VCG 2A and 4B isolates only caused mild to moderate external symptoms without defoliation [7,61,77,78]. Additionally, VCG 1A isolates were more virulent than VCG 2B isolates. VCG 1A isolates designated to a defoliating pathotype incited complete defoliation of inoculated cotton [7,79], whereas VCG 2B isolates induced partial defoliation of inoculated cotton and were thus designated as a defoliating-like pathotype [60]. However, in Australia, the non-defoliating pathotype (VCG 2A) caused more widespread and destructive disease in cotton than the defoliating pathotype (VCG 1A) [28]. Fields infested with *V. dahliae* VCG 2A can experience substantial yield loss of up to 50% (Figure 3). Additionally, pathogenicity tests determined that there was significant variability in virulence among isolates characterised as non-defoliating pathotype [80].

The virulence of *V. dahliae* is likely more related to its host of origin. For example, *V. dahliae* isolates originally from cotton were more virulent to cotton than to other crop hosts in many cases [77,81,82]. Similar observations were also reported on host crops, including cauliflower [81], peppermint [83], and lettuce [82]. However, many authors reported that the pathogenicity of *V. dahliae* was irrespective of its host of origin. Bao et al. [77] found *V. dahliae* isolates originally from cotton and eggplant were equally virulent to both hosts. Defoliating isolates of *V. dahliae* from cotton and artichoke also displayed comparable virulence to the two hosts [84]. Bhat and Subbarao [82] also found one cotton–*V. dahliae* isolate expressed host specificity to cotton among the 14 tested host crops. On the other hand, cotton–*V. dahliae* isolates that were non-pathogenic to cotton were also reported [58,77]. In many cases, isolates of *V. dahliae* were determined to be non-pathogenic based on external foliar symptoms and vascular discolouration of woody tissues [85,86]. However, symptomless infection of *V. dahliae* on some hosts may serve as inoculum reservoirs for subsequent crops [39,87,88].

On the whole, it is difficult to draw conclusive relationships between the virulence of *V. dahliae* populations and their VCG/pathotype classifications, as well as to its host of origin. Insights into the pathogenicity of specific *V. dahliae* populations will significantly contribute to developing better disease management programmes.

## 4. Detection and Its Advances

### 4.1. Culture-Based Techniques

Field-based symptoms for the detection of VW of cotton can lead to misidentification with Fusarium wilt due to overlapping symptomology. Therefore, isolation of the actual wilt pathogen(s) is always required. Though *V. dahliae* can be recovered from diseased plants using a common growth medium such as PDA, more than 20 semi-selective to selective media have been developed and modified for the accurate detection and enumeration of *V. dahliae* [89]. Only selected media are presented in Table 1. Ethanol agar was first developed by Nadakuvukaren and Hoener [90] for the enumeration of *V. dahliae* in soil, but the medium yielded a low amount for microsclerotial recovery [89,91,92]. The medium was then modified and recently, Mansoori [92] reported that ethanol potassium amoxicillin agar (EPAA) could recover up to 98% of microsclerotia in soil. EPAA is an ethanol-based medium with the amendment of potassium salts and replacement of streptomycin with amoxicillin. Soil extract agar was developed by Menzies and Griebel [93] and followed with many modifications. However, Mpofu and Hall [89] found soil extract was not essential for recovery of *V. dahliae* from soil. Of these, Sorensen’s NP-10 was a common selective medium used for the soil enumeration and recovery of *V. dahliae* from infected materials [7,11,39]. Sorensen’s NP-10 provided very good recovery of propagules that were clearly distinguishable when viewed under the microscope [94]. It was noticed that in all media, altering the components slightly can have significant impacts on the recovery of *V. dahliae*. For example, polygalacturonic acid (PGA) was an important component of Sorensen’s NP-10 media for quantifying *V. dahliae* in soil, but not all types of PGA equally favoured the growth and recovery of *V. dahliae* [94].

Soil plating, including wet and dry soil plating for the quantification of alive *V. dahliae* population, is laborious and time consuming compared to molecular approaches. However, it does not require expensive molecular equipment. The most effective semi-selective media and techniques should be determined by individual laboratories based on the skill level of diagnostic technicians using a microscope and the equipment available in their facility. Important considerations when selecting a method include spatial variation within fields, variability within replicates, variability within subsamples, and colony overlap within Petri plates due to the amount of soil applied to plates.

### 4.2. Culture-Independent Techniques

Advances in technology have allowed for the more rapid and accurate detection of *V. dahliae* [98,99,100,101,102]. A number of species-specific molecular markers were designed to accurately detect and differentiate *V. dahliae* from other species [101,103,104,105]. Due to the presence of non-defoliating and defoliating pathotypes, of which the latter has been believed to be more virulent and cause possibly higher yield losses [23,79], additional primers were designed to employ in multiplex PCRs for simultaneous differentiation of the two pathotypes [100,106]. A list of primers is shown in Table 2.

In addition to the rapid and accurate detection of *V. dahliae* both in vitro and in planta, some of those markers provide more qualitative information [98,102,112]. To some extent, detection and quantification of the *V. dahliae* population will assist in developing risk matrices for disease prediction and management. Paplomatas et al. [114] reported that at a population density as little as 1 microsclerotium per gram of soil, *V. dahliae* can cause VW symptoms of around 10% of cotton plants in the field. At a density of approximately 19 microsclerotia/g soil, VW incidence of cotton can be as high as 50% [24]. However, with recent soil sampling directly from the cotton rhizosphere, it was estimated that only about 11 microsclerotia/g soil could result in 50% wilt incidence [115]. Wei et al. [22] also found that *V. dahliae* inoculum thresholds varied depending on the susceptibility of cotton cultivar. Therefore, diverse cotton cultivars are grown in different ranges of ecological niches, and it is always desirable to adapt these referring findings for development of regional-specific inoculum thresholds. In cotton, the use of certified and *V. dahliae*-free seeds could prevent *V. dahliae* introduction into new growing areas [7,11]; however, assessing for *V. dahliae*-free seeds still relies on laborious and time-consuming plating assays using selective media. Newly developed LAMP-CRISPR/Cas12 and RPA-CRISPR/Cas12 were reported for their ultrasensitivity, being able to detect a single conidium within 90 min [105,113]. Therefore, these can be deployed for the certification of cotton seeds, although further research is required.

In-field quantitative cotton VW detection and surveillance are labour-intensive in addition to the field expertise requirements. In recent years, remote sensing technology has been developed and validated for the rapid field-based monitoring and quantifying of cotton VW severity in large areas [99,116,117,118]. From analyses of aerial images retrieved from narrow-band multispectral, hyperspectral, and thermal cameras, Li et al. [117] found significant correlations between VW incidence/severity of cotton crops, chlorophyll content, and leaf reflectance. The chlorophyll content and leaf reflectance index decreased as wilt symptoms progressed [99,117,118]. Advances in remote sensing technologies increased the confidence and accuracy of cotton VW detection in the field to over 90% [99]. Therefore, the technique could be a practical replacement for the early and accurate detection of VW in cotton fields. However, the accuracy of the technique is challenged in cotton fields where Fusarium wilt and VW co-occur [25,31].

## 5. Control Strategies

### 5.1. Chemical Strategies

Chemicals registered for the control of soilborne diseases are very limited, except for a few that can suppress the soilborne pathogen populations. The controlling of VW traditionally relied on soil fumigation to reduce viable inoculum, and subsequently reduced disease incidence and severity. Chemical fumigation is adopted widely to disinfest *V. dahliae*-infested soils growing high-value crops in horticultural and floricultural industries [119,120,121]. However, soil fumigation is not practical for broad-acre crops including cotton due to the high operation costs associated with the application [120,122]. Other alternative approaches, including soil solarisation, biological control, and organic amendment, have also been assessed for their soil disinfestation efficacies against *V. dahliae*, which will be discussed in later sections. Chemical application to control VW of cotton was evaluated by Kurt et al. [123]. Three applications at 21-day intervals of prochloraz (Sportak 45% EC, 450 g/L) and prochloraz–manganese complex (Sporgon 50 WP, 46% *w*/*w*) at high dosages of 506 g/ha and 1250 g/ha, respectively, decreased the VW disease severity of cotton grown on naturally infested soil [123]. The findings provide an additional potential approach for integrated disease management.

### 5.2. Cultural Strategies

Cultural practices such as crop rotation, organic amendments, irrigation and nutrient management, and quarantine are commonly deployed in cotton fields to control VW. These practices were designed to (1) reduce *V. dahliae* density and improve soil health by altering host crops and soil microbe community and improving organic matter inputs [124]; and (2) contain and minimise further spread of *V. dahliae* to unaffected fields [125]. It is theorised that in an absence or with a fewer number of cultivated hosts, the soil population of *V. dahliae* will decrease. However, *V. dahliae* produces microsclerotia that can survive for many years without the presence of hosts [35,36]. Therefore, the reduction in *V. dahliae* population will be dependent on many factors, including the occurrence frequency of host and non-host crops. Additionally, cotton has a low microsclerotia threshold that can result in VW symptoms and yield loss [22]. Wheeler et al. [126] reported that VW of cotton was managed by short crop rotations for only four years after the first VW symptoms occurred. Similarly, one-year rotation with paddy rice or perennial ryegrass [127], or with maize and broccoli [124], reduced the VW incidence significantly. However, after 11 years of VW, the wilt incidence and yield in every two-year crop rotation plot were comparable to the continuous cotton plot [126]. *Verticillium dahliae* has a wide host range, including many weed species and non-hosts [10,38,39,40,128]. Therefore, the effectiveness of crop rotation in managing VW of cotton remains highly challenging and dependent on non-host selection and successful weed management [40,129,130]. According to Huisman and Ashworth [131], rotations stretched out the *V. dahliae* inoculum buildup but were unable to prevent infection. However, rotations did provide some agronomic benefits, including altering soil microbe communities and improving soil physicochemical properties that can subsequently increase yields [124,131]. Zhao et al. [124] observed that NO_3_^−^-N contents were increased in cotton–maize (CM) and cotton–maize–broccoli (CMB) rotations in comparison to cotton–cotton (CC) cropping. Similarly, microbial abundance and composition under the CM and CMB rotations were significantly different from those of the CC cropping system [124,132].

In addition to crop rotation, organic amendments are commonly practised to improve the soil properties that can suppress VW in cotton fields [133]. Huang et al. [133] reported that soil amendments with crab shell (2% *w*/*w*), soybean stalk, and alfalfa (1% *w*/*w*) increased rhizosphere microbes, including significant antagonists against *V. dahliae*. Subsequently, VW severity was reduced up to 72% relative to the control. Similarly, the application (1% *w*/*w*) of bio-organic fertiliser containing amino acid, manure compost, and *Bacillus subtilis* stimulated the development of unique beneficial fungal groups [134,135]. VW incidence was recorded at 4.4% in comparison to 90% in control [134]. However, the efficacy of organic amendments highly varied depending on soil properties [136]. According to Ochiai et al. [137], organic amendment-based systems for VW management are hardly realistic in broad-acre cropping, since it is challenging to achieve and retain an appropriate level of disease suppression.

Along with organic matter inputs, irrigation and nitrogen inputs are also important to achieve targeted yield in cotton crops [8,20,138]. However, it was a trade-off between providing sufficient inputs to maximise yield and profit but at the same time not promoting excessive disease development [20]. Wheeler et al. [20] reported that the highest yield occurred with a base irrigation rate, where the irrigation was undertaken to meet 80% of the crop needs. Unfortunately, the VW rating was higher in comparison to the irrigation-reduced (half-rate) treatment. Increasing the irrigation rate to 1.5 times the base rate resulted in the highest level of VW [20,138]. On the other hand, the impact of nitrogen inputs on VW and yield was unclear [20,139]. However, excessive irrigation and nitrogen inputs may result in more VW and damage in cotton [140].

After harvesting, cotton stubbles were slashed and returned to the soil. Zhang et al. [141] demonstrated that stubble return was important in improving soil microbial community as well as C and N contents, which can help to improve soil health. However, in fields where VW occurred, stubble return means returning of the inoculum in the soil and increases risks of VW occurrence in the following years [141]. Zhang et al. [142] reported that the number of microsclerotia increased from 0.16 to 8.37 times in rhizosphere soil that received the *V. dahliae*-infected stubbles. In Australia, it was recommended to slash and incorporate cotton stubbles as soon as possible after harvesting to provide a host-free period and stimulate decomposition [143]. To some extent, cultural practices may reduce the VW level and yield impacts. Unfortunately, in cotton fields heavily infested with *V. dahliae*, the overall profitability was reduced [20].

*Verticillium dahliae* is a soilborne pathogen and can be introduced from one field to another via movements of soil-covered footwear and farm machinery. In Australian cotton farming systems, a ‘Come Clean Go Clean’ policy was widely adopted to minimise the risk of further spread of soilborne pathogens [144]. Growers rely on disinfectants to wash off and decontaminate their farming equipment. Nguyen et al. [125] reported that the efficacy of tested disinfectants was greatly dependent on treatment time and *V. dahliae* spore types, which were not mentioned on the disinfectants’ labels. On-farm hygiene practices must be reinforced and reconsidered to improve effectiveness in decontaminating farming equipment, and subsequently, to minimise risks of further introduction of *V. dahliae* to new fields and properties.

### 5.3. Biological Strategies

The biocontrol of *V. dahliae* in cotton is well researched. A number of rhizosphere bacteria and endophytic fungi were recovered and assessed for their biocontrol efficacy against VW of cotton (Table 3). The efficacy of these potential biocontrol agents in protecting cotton from *V. dahliae* infection varied greatly from 32.5% to 93.6% in pot trials [145,146]. Meanwhile, under field conditions, the protection efficacy varied from 28.4% to 76.4% [147,148]. The control efficacy was dependent on application methods and number of applications. For example, pre-inoculation of *Gibellulopsis nigrescens* (previously known *V. nigrescens*) recovered from cotton plants with VW symptoms protected cotton seedlings in pot trials from subsequent inoculation of pathogenic *V. dahliae*. Disease incidence and severity were reduced by up to 95% and 97%, respectively. However, co-inoculation of the two fungi at the same time reduced the level of protection by *G. nigrescens* up to 60% [149]. Cotton seedlings (two to three emerging leaf stages) drenched with 10^9^ CFU/mL of *Enterobacter cancerogenus* HA02 reduced the VW incidence by 80% in pot trials, while the field applications were required to be repeated every 15 days to achieve 50% protection [150].

Biological control is believed to be a sustainable and environmentally friendly approach. However, there is yet to be a registered biocontrol agent against *V. dahliae*. Successful biocontrol agents at the field scale need to satisfy all of the following criteria: (a) reduce pathogen population in the soil bank; (b) reduce disease incidence on a subsequent crop; (c) improve yields of infected plants to levels comparable with healthy plants or crops grown in pathogen-infested soils [162,163,164]. Additionally, some other challenges, including large-scale production, formulation and preservation conditions, and application methods, should be considered during a long-term selection process [163].

### 5.4. Induced Resistance

Controlling VW of cotton relies on effective integrated management and the adoption of resistant cultivars. However, none of the cultivated cultivars provided complete resistance to *V. dahliae* [165,166]. In cotton, a number of biological agents and chemistries were reported to enhance natural resistance, known as induced resistance, against *V. dahliae* [161,167,168]. Hansan [161] found cotton grown from *Trichoderma virens*-coated seeds had significantly lower wilt severity compared to untreated seeds. However, *T. virens* did not provide direct antagonistic activities towards *V. dahliae*. Therefore, the protection provided from the *T. virens*-treated seeds was proposed to relate to induced resistance [161,169]. Howell [169] discovered that cotton roots colonised by *T. virens* induced a higher level of terpenoid synthesis, which was correlated to *Verticillium* resistance in cotton. A by-product, mycelial mass of *Penicillium chrysogenum* from pharmaceutical industry, was also reported for its induced resistance capability against VW of cotton [168,170]. Cotton grown in a potting mix amended with 2% of dried mycelia of *P. chrysogenum* had a significantly lower disease severity compared to that of the control. The percentage of protection was up to 53%. A similar control efficacy was also observed on cotton drenched with 5% of the water extract of dry mycelia [170]. Neither the dried mycelia nor its water extracts were inhibitory to the growth of *V. dahliae*. Levels of peroxidase activities, which played roles in wilt resistance [171], were increased significantly in cotton treated with both the dried mycelia and its extract [170,172]. Chen et al. [173] later found that dried mycelia of *P. chrysogenum* was capable of inducing resistance in treated plants through activating the salicylic acid pathway, which was also cross-talking with the jasmonic acid pathway. Similarly, *P. aurantiaca* ST-TJ4 and *Bacillus altitudinis* KRS010 were also discovered to induce VW resistance in cotton [146,174].

Foliar application of benzo (1,2,3)-thiadiazole-7-carbothioc acid S-methyl ester (BTH) after appearance of the first wilt symptom reduced the disease severity of field cotton by approximately 35% compared to unsprayed control [167]. BTH is a well-known plant activator and proven to enhance natural resistance on numerous crops [175]. The BTH control efficacy toward VW of cotton was probably due to induced resistance, since there was not a direct inhibitory effect of BTH against *V. dahliae* [167,176]. However, the associated inducing pathways were still uncertain [176]. It is clearly indicated that VW resistance of cotton can be enhanced both under experimental and field conditions by inducing its natural resistance. Therefore, exploiting some other plant activators for their induced resistant potentials against VW of cotton could be valuable, pending the discovery of highly or complete resistant cultivars.

### 5.5. Host Resistance

One of the most effective and economical control measures for VW is the use of resistant cultivars [29]. In the interaction between VW and cotton, as well as with other plant species, the term resistance is preferred instead of tolerance. Molecular and histopathological observations showing that infected plants activate mechanisms to delay and restrict pathogen colonisation in the vascular tissue have shaped current definitions of resistance [177,178,179,180] and have recently been expanded by new insights into signal transduction, immune response pathways, and transcriptional regulation [181].

#### 5.5.1. Mechanisms of Resistance

When cotton plants are infected with *V. dahliae*, there are a wide range of physical and/or biochemical resistance mechanisms that can be either present constitutively or actively deployed [182]. The interaction with the environment, timing and extent of the deployment will determine the level of resistance of a given cotton genotype. Non-host immune responses to this disease were not found in cotton [52], and therefore resistant plants are also infected but show limited colonisation in their roots or other tissues by the fungus [180,181].

Cotton plants activate physical resistance mechanisms upon infection by *V. dahliae*. This includes the formation of tyloses in resistant genotypes to block stem vessels, thereby limiting pathogen spread [183]. While some studies note similar defence strengthening (*G. barbadense* developing callose and cellulose in cell walls) occurring later in susceptible plants [178], other research highlights a rapid, coordinated response. Specifically, resistant genotypes show extensive and rapid accumulation of lignin, phenolics, callose, and reactive oxygen species [180,184]. This accumulation, often linked to increased expression of lignin synthesis genes, underscores that a swift physical response is critical for effective resistance [179].

Physiological and biochemical mechanisms are very important in the defence reaction of cotton plants against *V. dahliae*, normally in combination and synchronised with the physical mechanisms mentioned before [185]. Antimicrobial compound accumulation, including phytoalexins, phenolics, and tannins, contributes to fungal suppression and vascular discoloration [180]. Recent transcriptomic data show rapid activation of hormone signalling and reactive oxygen species (ROS) regulation upon infection [181]. These cascades are tightly integrated and appear to act synergistically across tissues and time points. Limited work has elucidated the specificity of defence mechanisms against *V. dahliae*; however, numerous studies have identified the associated resistance genes and pathways [186].

#### 5.5.2. Sources and Inheritance of Resistance

Cultivated upland cotton is generally highly susceptible to VW, and no immunity to the disease exists in this or the other three cultivated species in the *Gossypium* genus. Among these, *G. barbadense* remains the most resistant, followed by *G. arboreum* and *G. herbaceum* [165,166,187]. Many other wild *Gossypium* diploid species, such as *G. raimondii* and *G. stuartinum*, show high levels of resistance to VW [188,189] although their use remains limited due to reproductive barriers and genetic distance from cultivated lines. The incorporation of resistance traits is a long and sometimes complicated process and can be approached with non-traditional techniques such as the development of synthetic amphiploids [184].

Resistance sources differ in the mechanisms they activate, which may reflect different combinations of physiological and biochemical mechanisms [190,191]. In *G. barbadense*, resistance is controlled by a few major genes [165,192], while in *G. hirsutum*, resistance seems to be polygenic with low to moderate heritability, controlled by at least two major genes and some additional ones [193,194,195]. The inconsistency among the different authors classifying resistance and its inheritance was later confirmed with molecular studies (see below) and suggested that VW resistance in cotton is generally a polygenic trait, and therefore highly dependent on factors such as virulence of the *V. dahliae* pathotypes [195], and the temperature, growth, and development of the plants. These factors, together with the specific plant population used, modify the resistance reaction obtained and therefore the inheritance pattern observed [194,196].

#### 5.5.3. Genetics of Resistance and Mapping of Quantitative Trait Loci

In other crops such as tomato, major genes providing resistance to VW have been identified [197]. This resistance gene also was demonstrated to be effective against the same *V. dahliae* race 1 in *G. hirsutum* plants that had the gene via transformation [198]. Using a homologous gene approach, several Ve-like resistance genes have been identified in cotton. *GbVe* and *Gbve1*, isolated from *G. barbadense*, are the closest analogues of a true resistance gene found in cotton, and have been functionally validated in Arabidopsis to confer resistance to both defoliating and non-defoliating isolates [165,199]. A third gene, *Gbvdr3*, also enhances resistance but specifically to defoliating pathotypes [184]. Another gene, *GbaNA1*, appears to confer resistance to non-race 1 isolates, suggesting the involvement of recognition pathways independent of Ave1 [200].

Additional genes and gene families contributing to resistance have been identified through transcriptomics, expression analysis, and functional validation. These include candidates involved in hormone signalling, lignin biosynthesis, ROS detoxification, and receptor-like kinases [201,202,203,204,205,206,207]. However, in *G. hirsutum*, resistance appears to be more polygenic and less reliant on single major genes. While early studies identified some resistance-related genes in specific cultivars such as Sicala V-1 [208], more recent genome-wide studies have greatly expanded this list [186].

Similarly, more than 200 QTLs partially explaining VW resistance have been detected, using different types of markers, in nearly every single cotton chromosome in a range of *G. barbadense* and *G. hirsutum* genotypes. However, some ‘hotspots’ with a higher occurrence of QTLs were identified in a more reduced number of chromosomes [209,210]. This finding also supports the polygenic and complex nature of VW resistance in cotton, which currently limits the application of the identified QTLs in breeding programmes [211]. Nonetheless, there have been some attempts at introgressing VW resistance using marker-assisted selection. For example, Li et al. [212] screened a number of SSR markers previously reported to be linked to VW resistance. They found five markers which significantly linked to a higher resistance and a combination of two of them which should be given preference when performing marker-assisted selection. In another study, the resistance level of *G. hirsutum* was successfully increased in controlled conditions by using the gene-editing technique to edit two copies of the transcription factor regulator gene *Gh14-3-3d* [213].

In a GWAS combined with QTL-sequence and transcriptome analysis, Zhao et al. [214] identified eight candidate genes related to basal defence mechanisms, flavonoid biosynthesis, and transcriptional regulation in *G. hirsutum*. These genes were associated with significant SNPs and validated using KASP markers. Similarly, using four recombinant inbred line (RIL) populations, Wang et al. [215] identified eight QTLs across four chromosomes, including a novel and stable QTL, qVW-A12-5, which contains the Gh_CPR30 gene, functionally validated via gene silencing. Recombinant inbred line (RIL) populations have been used to dissect these resistance loci. A recent study identified major resistance QTLs in RILs derived from a cross between resistant MCU-5 and susceptible Siokra 1–4, with transcriptome analysis revealing 99 differentially expressed genes in these regions [216]. These findings reinforce the polygenic nature of resistance and highlight the importance of integrating genomic tools in breeding programmes.

#### 5.5.4. Breeding for Resistance

High resistance to *V. dahliae* is rarely found in *G. hirsutum*, and most breeding efforts have therefore focused on introgression from resistant species such as *G. barbadense*, *G. arboreum*, and from elite resistant lines. In the early 1900s, the Mexican landrace ‘Acala’ was introduced in the USA to improve fibre quality and became the basis of resistant Acala cultivars. As new *V. dahliae* pathotypes emerged, resistance was enhanced through backcrossing, reselection, and introgression from *G. barbadense* [196]. In the 1970s, novel resistance sources were added via the San Joaquin family, derived from *G. arboreum* and *G. thurberi*. These and other resistant *G. hirsutum* sources, including landraces from Mexico, formed the basis for varieties later developed in Australia and the CIS region [52]. Additional resistance from wild diploid species was also incorporated [189]. However, some programmes showed limited progress—for instance, varieties released by the Oklahoma Agricultural Research Station during the years 1918–1982 exhibited modest levels of resistance [217].

Over the last 40 years, the level of resistance has been maintained or slightly increased generally by new introgressions from *G. barbadense* [165], and extensive efforts are being made to increase the level of VW resistance [165,218]. For a long time, plant-breeding programmes located in areas where the pathogen was widespread have been indirectly breeding for VW resistance and other diseases when breeding for better yield performance [219]. Such is the case for Australia, where all cultivars developed since the release of Sicala V-1 and V-2 in 1991 and 1994 are among the most resistant cultivars worldwide [18]. Recent studies have advanced breeding by identifying candidate defence-related genes in resistant plants [216,220]. These discoveries offer promising targets for marker-assisted and genomic selection in *G. hirsutum* breeding.

#### 5.5.5. Screening and Selection Methods

The expression of resistance to *V. dahliae* in cotton results from a complex interplay between genetic background, environmental conditions, and pathogen pressure. While genetic resistance forms the basis of any breeding effort, the reliability of phenotypic assessment is tightly linked to where and how resistance is evaluated—whether in infested field conditions, controlled environments, or through alternative indirect approaches.

**Figure 4:**
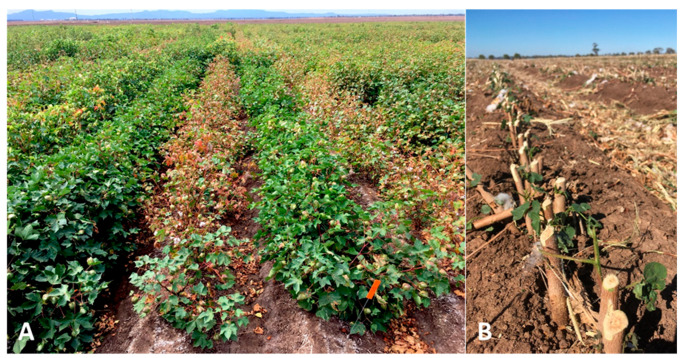
Evaluation of cotton genotypes for resistance to VW in field conditions. (**A**), experiments with replicated plots showing cultivars with different severity levels of foliar symptoms caused by *V. dahliae*. (**B**), Evaluation of disease incidence by quantifying vascular browning in stems infected by *V. dahliae*.

Historically, screening for VW resistance in cotton began in the 1940s using heavily infested soils in the United States [52]. These field trials provided a realistic context to observe resistance under natural pathogen populations and climatic variation. However, soil inoculum distribution is often highly variable and symptom expression highly inconsistent [22,115]. For population-level comparisons involving large numbers of plants and replicates, these environments are valuable; yet they prove less effective when attempting to select individual plants. Reference cultivars with known resistance levels become essential to calibrate evaluations across seasons, field sites, and management systems. Typically, resistance assessments rely on the visual scoring of vascular browning and foliar symptoms (Figure 4), timed during boll development to avoid confounding disease symptoms with late-season senescence [196,221]. High disease incidence generally leads to yield loss unless infection occurs late in the crop cycle [193], and while yield can serve as an indirect selection trait, comparisons must be limited to genotypes expressing similar symptom levels [18]. Additionally, many factors can modify the level of resistance observed under field conditions. Among them, symptoms and yield loss were reported to increase significantly with higher crop load, lower plant densities [19,222], the presence of root-knot nematodes [221], and the application of high irrigation and nitrogen rates [20].

To improve precision, resistance screening often shifts to controlled conditions, where inoculation is standardised. In greenhouses or growth chambers, young plants are typically inoculated via root dip or stem injection [165,223]. This approach shortens evaluation cycles and permits high-throughput phenotyping with relatively low resource demand. Still, translating resistance performance across conditions is not always straightforward. While field and greenhouse ratings often correlate [187,218,224], the inoculation method can influence outcomes [223]. In greenhouse studies, a single inoculation using a conidial suspension is typical [196], whereas in the field, infection is continuous and may involve microsclerotia in soil. To bridge this gap, some researchers have employed natural or artificially infested soils in pots, allowing for a more field-like infection process [225]. These methods are slower and less scalable but yield insights into the relationship between inoculum density and disease severity.

Other discrepancies are tied to environmental variation. For example, field temperature fluctuations and sun exposure can activate resistance-related compounds like tannins [226]. Some resistance traits, such as the ability to develop extensive root systems that escape infection zones, go undetected in pot-based assays [227]. Likewise, greenhouse-selected lines can show unexpected susceptibility under field nutrient limitations, particularly potassium [228]. In both field studies and those under controlled conditions, temperature and inoculum (density and virulence), and the interaction between them, are the most critical parameters to evaluate cotton genotypes for resistance to VW. Bell and Presley [229] demonstrated that resistance expression shifts markedly with just a 4 °C temperature change. A genotype classified as moderately resistant at 29 °C might appear susceptible at 25 °C.

In parallel with traditional phenotyping, where disease progression is usually monitored using severity scales, new technologies have begun to reshape early-stage screening. For instance, high-resolution hyperspectral imaging coupled with machine learning has shown strong promise in detecting asymptomatic infections in cotton with over 90% accuracy [117,230]. While not a replacement for classical methods, these tools offer non-destructive, scalable alternatives that can accelerate selection, particularly when symptom development is inconsistent.

### 5.6. Integrated Strategies

There are no completely resistant cultivars available; therefore, controlling VW of cotton cannot rely on any single management strategy. Soil solarization was trialled in fields infested with *V. dahliae*; however, this was an expensive approach for soil disinfestation and perhaps impractical for large-scale use in cotton. However, it can be applied for disease suppression in field hotspots [231]. The population of *V. dahliae* in plots covered with thin transparent plastic film (25–37 µm thick) and solarized for 6–10 weeks was very low or undetectable and, subsequently, the incidence of cotton wilt was reduced to 13% compared to 55–90% in un-solarized plots. Additionally, VW of cotton was delayed by 2–7 weeks in treated plots, but this is not a practice that is viable economically and in the long term, since the *V. dahliae* population bounced back sharply in plots followed by planting with susceptible cultivars. This practice was recommended for an integrated approach with crop rotations and use of resistant cultivars [16].

A synergistic effect in reducing *V. dahliae* viability was observed in a combined treatment of solarization and metham sodium. Metham sodium alone at 25 mL/m^2^ reduced the viability of *V. dahliae* by 70% compared to an untreated control after a week of treatment. In comparison, the *V. dahliae* population was only reduced by 3% in solely solarized plots after a week of solarization. With the same treatment duration, there was no detection of *V. dahliae* in plots treated with solarization plus metham sodium at 25 mL/m^2^ [232].

Soil populations of *V. dahliae* were significantly decreased in plots amended with either broccoli or ryegrass biomass and covered with airtight plastic films, compared to untreated, organic amendment only and plastic cover only [233]. This potential approach can be an alternative practice for soil disinfestation where chemical strategies, solarizing, and flooding are not feasible [233]. Anaerobic soil disinfestation manipulated by applying rice bran (17–20 t/ha) and water saturation for 3–6 weeks effectively protected strawberry plants from *V. dahliae* up to 100% in field conditions. Net economic returns were equivalent to those from chloropirin fumigation. Strawberry farmers are currently adopting the practice [234].

The potential of Brassica crops to reduce *V. dahliae* density were evaluated. Nineteen cultivars were selected and screened for their control efficacy against *V. dahliae* when amended in soils. The mortality of *V. dahliae* in naturally infested soils varied from 9 to 90% depending on soil type and cultivars tested [235]. Microsclerotia of *V. dahliae* in soil amended with 0.4% *v*/*v* of defatted seed meals (BioFence^TM^, Cobham, Surrey, UK) of either *B. juncea* or *B. carinata* was completely eliminated in in vitro assays conducted on artificially inoculated soil. However, the efficacy was reduced by 20–80% when these were amended into naturally infested soil (4 t/ha). The efficacy was greatly dependent on soil types and better control effect was recorded on sandy soil with low carbon contents [236]. Glucosinolate and isothiocyanate released from the brassica meals were believed to be toxic to *V. dahliae* [236]. Another individual assay with BioFence^TM^ derived from *B. carinata* recorded a reduction in *V. dahliae* density in naturally infested soil of only 27% compared to an untreated control [237]. These provide alternative practices to suppress the *V. dahliae* population, but they were not sufficient enough to eliminate the VW risk for some of the sensitive crops [237].

## 6. Future Perspectives

It is of no doubt that VW is a disease of significance in cotton worldwide. The disease can be managed, which relies on surveillance and accurate identification using field-based symptoms and further confirmation with molecular techniques such as PCR, RPA, and LAMP [101,105]. Currently, a total of 109 genome assemblies are available across 11 *Verticillium* species, with more than half (i.e., 58 genomes) being *V. dahliae*. These genomes are great resources for comparative genomics analyses to identify species-specific genes that could enhance the specificity and sensitivity of molecular detection assays of *V. dahliae* [104,238]. Additionally, high-throughput sequencing (HTS) has become more accessible and cost-effective these days for rapid and accurate identification of plant pathogens, especially those that are emerging [239]. However, HTS datasets and genomic analyses are not universally accessible, since they require complex bioinformatic tools, expertise, and knowledge for meaningful interpretations [239]. Therefore, species-specific markers remain highly important for *V. dahliae* detection and surveillance. Emerging applications of artificial intelligence in genomic analyses could assist in optimising identification and selection of target genes for marker development [240].

*Verticillium dahliae* has a wide host range and is mostly associated with its VCG designations and host of origins. Non-pathogenic isolates of *V. dahliae* were also reported in cotton. However, it was difficult to interpret the relationship between the virulence of *V. dahliae* population and its VCG/pathotype designations on many occasions. Therefore, it is always important to study the pathogenicity of each specific *V. dahliae* population, and this will facilitate the development of better cropping systems for disease management. The increasing available genomic resources of *V. dahliae* allowed for the identification of biosynthesis gene clusters associated with virulent mechanisms [201,241]. For example, deletion of two mitogen-activated protein kinases showed altered responses to osmotic stress, fungicidal response, and cell-wall stressors with virulence completely abolished for one of them (strain ΔVdSte11) due to its failure to penetrate the cell wall and form hyphodopia in *V. dahliae* [242]. These results suggest that genes involved in microsclerotia formation are important for its pathogenicity. Additionally, lineage-specific regions of the *V. dahliae* genome, that are often enriched with effector genes, appeared to show conserved sequences in both coding and non-coding regions [243]. Genomic survey using short-read sequencing on isolates collected in major potato-producing regions of Canada revealed an abundant level of genetic variation and two major lineages of *V. dahliae* strains that infect potato [66]. Comparative genomic studies have shown that extensive genomic rearrangements have occurred during *Verticillium* evolution, leading to gene losses [201]. Furthermore, systematic search for horizontal gene-transfer events in the genome of *V. dahliae* suggested possible horizontal gene acquisition from *Fusarium* [201].

Genomics so far have provided insights into effector repertoires and signalling pathways in *Verticillium* species. This knowledge can accelerate the development of fungicides by targeting specific effectors in *Verticillium* [244,245,246]. For example, lineage-specific regions in the genomes of different *V. dahliae* races showed structural variations and through comparative analysis, may explain specific secreted proteins that could determine the virulence among these strains [247]. Using reverse genetics, a virulence factor, VdEPG1, from the Glycoside hydrolase family, can suppress programmed cell death by modulating pathogenesis-related genes in tobacco, and its deletion led to reduced pathogenicity of *V. dahliae* in cotton [248]. *V. dahliae* transcription factors Som1 and Vta3 have been shown to control microsclerotia formation and participate in root penetration in Arabidopsis [249]. Through transcriptome sequencing, it was shown that Vta3 alters gene expression of other virulence factors and leads to transcriptome reprogramming of certain gene networks for late stages of plant disease progression [250].

Host-induced gene silencing (HIGS) has emerged as a potential strategy for the management of *V. dahliae* in various plant hosts [251]. In cotton, thiamine transporter proteins are modulators of pathogenesis [251]. By using HIGS to introduce double-stranded RNA (dsRNA) targeting these genes, it was shown that the resulting transgenic cotton plants produced enhanced disease resistance to *V. dahliae* and yield compared to the WT lines in the field [251]. In a similar study, dsRNA knockout mutant was generated for a chitin deacetylase gene from *V. dahliae*, and its knockout severely reduced spore production and penetration [252]. This led to the development of an RNAi-based nanopesticide to control *V. dahliae*, offering a novel strategy to manage VW [252].

To sum up, genomic research will continue to advance our understanding of *V. dahliae* biology, diversity, evolution, and pathogenicity, that consequently assist in disease management and breeding strategies. Until a cotton cultivar with complete resistance to *V. dahliae* becomes available, the management of VW of cotton requires the use of multiple complementary tactics. The ongoing research into resistant resources will continue to improve the VW resistance level in cotton [165,218]. Future research may focus on GE-cotton to accommodate VW-resistant genes [182].

## Figures and Tables

**Table 1:** Summary of recipes of some semi-selective and selective media used for isolation and quantification estimation of *V. dahliae* from infected plant materials and soils.

Media	Constituents for 1 L of Medium	References
Ethanol agar	Ethanol (0.5 mL), streptomycin (100 mg), agar (7.5 g)	[90]
Modified ethanol agar	Ethanol (5 mL), sucrose (7.5 g), KCl (0.5 g), K_2_HPO_4_ (1 g), NaNO_3_ (2 g), MgSO_2_·7H_2_O (0.5 g), FeSO_4_·7H_2_O (0.01 g), streptomycin (100 mg), chloramphenicol (250 mg), agar (20 g)	[91]
Ethanol potassium amoxicillin agar (EPAA)	Ethanol (2 mL), KH_2_PO_4_ (1.5 g), K_2_HPO_4_ (4 g), amoxicillin (50 mg), agar (15 g)	[92]
Soil extract agar	Soil extract ^1^ (25 mL), KH_2_PO_4_ (1.5 g), K_2_HPO_4_ (4 g), chloramphenicol (50 mg), streptomycin (50 mg), chlorotetracycline (50 mg), agar (15 g)	[93]
Modified soil extract agar	Soil extract agar medium, polygalacturonic acid (5 g), ethanol (0.5 mL), tergitol (0.5 g)	[95]
Sorbose agar	Sorbose (2 g), streptomycin (0.1 g), agar (10 g)	[96]
Sorbose asparagine agar	L-sorbose (2 g), L-asparagine (2 g), K_2_HPO_4_ (1 g), MgSO_2_·7H_2_O (0.5 g), quintozene (1 g), NaB_4_O_7_·10H_2_O (0.3 g), KCL (0.5 g), Fe-Na-EDTA (0.01 g), oxgall (0.5 g), agar (20 g)	[97]
Sorensen’s NP-10	Tergitol (0.5 mg), KH_2_PO_4_ (1 g), KNO_3_ (0.5 g), MgSO_2_·7H_2_O (0.5 g), NaOH (0.025 N), polygalacturonic acid ^2^ (5 g), chloramphenicol (50 mg), streptomycin (50 mg), chlorotetracycline (50 mg), agar (15 g)	[94]

^1^ Soil extract was prepared as follows: a mixture of 1 kg soil and 1 L water was steamed for 30 min, decanted the water and filtered. ^2^ Polygalacturonic acid from orange (P-1879, Sigma-Aldrich, St. Louis, MO, USA) was discontinued and replaced by P-3889 (Sigma-Aldrich, St. Louis, MO, USA).

**Table 2:** A number of molecular markers were developed for species-specific detection as well as the pathotyping of *V. dahliae* from infected plant materials and soils.

Primers	Sequences 5′-> 3′	Detection Assays	Targets ^1^	References
VMSP1	CATAAAAGACTGCCTAC GCCG	Simplex	*V. dahliae*	[107]
VMSP2	AAGGGTACTCAAACGGTCAG			
DB19	CGGTGACATAATACTGAGAG	Simplex	*V. dahliae*	[108]
DB22	GACGATGCGGATTGAACGAA			
Vd-F	GGGTAGGCTGGCCATATGTG	Simplex	*V. dahliae*	[104]
Vd-R	GTTCTATCCATCGCGGAAAC			
NDf	ATCAGGGGATACTGGTACGAGA	Simplex	ND pathotype	[109]
NDr	GAGTATTGCCGATAAGAACATG			
INTND2f	CTCTTCGTACATGGCCATAGATGTGC	Nested	ND pathotype	[109]
INTND2r	CAATGACAATGTCCTGGGTGTGCCA			
D1	CATGTTGCTCTGTTGACTGG	Simplex	D pathotype	[110]
D2	GACACGGTATCTTTGCTGAA			
INTD2f	ACTGGGTATGGATGGCTTTCAGGACT	Nested	D pathotype	[111]
INTD2r	TCTCGACTATTGGAAAATCCAGCGAC			
espdef01	TGAGACTCGGCTGCCACAC	Duplex with DB19/DB22	D and ND pathotypes	[100]
NEP_InPC4	GGGACTGGGACAGGATGGACA	Duplex	D and ND pathotypes	[106]
Dnep286_R	CAAGACCAAATTCGACAGGCAGAG			
NDnep482_R	CCTATTACGAGGTACTTACGGGGACTCTT			
VdUni-F	TCCTAGGCAGGCGAGCAG	qPCR	*V. dahliae* in soil	[112]
VdUni-R	TAGGGCTGTCTGTCGGTGA			
VertBt-F	AACAACAGTCCGATGGATAATTC	qPCR	*V. dahliae* in plant tissue	[98]
VertBt-R	GTACCGGGCTCGAGATCG			
VTP1-2F	CTCGATCGTCGTCAACC	qPCR	*V. dahliae* in plant tissue	[102]
VTP1-2R	TGGTGGTGAGAGTGT TG			
FIP	CGTGATGCTCCGTTTAGGTGGATTTTCGCCATGTTCGGTGCTAG	LAMP	*V. dahliae* in soil	[101]
BIP	TGGCACGTGTGGCGTAAGACTTTTCGATGTCGAGTCTGACACTG			
F3	TGGCAGCTTCTGATTCAGTT			
B3	ACAGCGATTTGGATTCCCTC			
LF	CTGACAACCAACGTCTAGATCTCA			
LB	GGCTATTGAGTTCTGCACTCTGTC			
RPA-F	CTTCATTGAGACCAAGTACGCTGTAAGTAACC	RPA-CRISPR/Cas12	*V. dahliae* in soil and plant tissue	[105]
RPA-R	CAGTTGTCGTGAAGGGGTCATCTTGACTGC			
crRNA1R	GAACCCCAGCACATGATAGAATCTACACTTAGTAGAAATTA			
ssDNA-FQ	5′FAM-/TTATTATT/-3′DBQ1			
VD-FIP	TCTCCGTGGATGTTCTCGGGAATAATGGCTGCCGTGACTGTC	LAMP-CRISPR/Cas12	*V. dahliae* in soil	[113]
VD-BIP	TAGGGACGCAACAATGAGCTGTGCACGGCGCCAAAGTTC			
VD-F3	AGCGGAAGGGGCACTAG			
VD-B3	CAAAGACCACGACCATAGGC			
VD-LF	ACGATTGGCAGTCACGGTT			
VD-LB	TTGACGGCTTTACCACAGTCT			
crRNA	UAAUUUCUACUAAGUGUAGAUCCACAGUCUUCUCGGCCAAGU			
ssDNA-1	(FAM)CCACGGGAGGAATACCAACCCAGTG(BHQ1)			

^1^ Detection targets of developed assays were specific to *V. dahliae* and/or to defoliating (D) and non-defoliating (ND) pathotypes.

**Table 3:** A list of potential biocontrol agents, including rhizosphere bacteria and endophytic fungi, that were recovered and assessed in vitro from a glasshouse (GH) and from a field for their control efficacy against *V. dahliae* in cotton.

Bio-Agents	Isolates	In Vitro Efficacy	In Planta Efficacy (GH and Field)	Plant Growth Promotion	References
*Bacillus altitudinis*	KRS010	74–80%	93.6% (GH)	Yes	[146]
*B. amyloliquefaciens*	YZU-SG146	68.2–89.2%	84.2% (GH)	Yes	[151]
*B. atrophaeus*	YL84	72.2–84.1%	66.7% (GH)	Yes	[152]
*B. mojavensis*	KRS009	90.2%	88.6% (GH)	Yes	[153]
*B. subtilis*	T6	63.8%	92.6% (GH)	Not tested	[154]
*B. subtilis*	KRS015	59.2–97.1%	62% (GH)	Yes	[155]
*B. subtilis*	SM21	48.2%	45.7% (GH)	Not tested	[148]
*Bacillus cereus*	AR156	67.3%	74.3% (GH)	Not tested	[148]
*Serratia* sp.	XY21	41.3%	61% (GH)	Not tested	[148]
Consortium	SM21:AR156:XY21	77.3%	86.1% (GH), 43.3–76.9% (field)	yes	[148]
*Enterobacter cancerogenus*	HA02	Not tested	72.4% (GH), 45.9% (field)	Yes	[150]
*Paenibacillus polymyxa*	ShX301	87%	40.3–71.1% (GH)	Yes	[156]
*Penicillium simplicissimum*	CEF-818	Not tested	41.5–60.7% (GH), 62.4–69.5% (field)	No	[157]
*Pseudomonas* spp.	FP22, FP23, FP30, FP35	43.9–56%	32.5–50% (GH), 22.1–50.9% (field)	Yes	[145]
*Streptomyces kanamyceticu*	B-49	68.9–85.6%	65.8% GH), 28.4% (field)	No	[147]
*Acremonium* sp.	CEF-193	Not tested	52.4–47% (GH), 38.5–54.6% (field)	No	[157]
*F. oxysporum*	By125	Not tested	69% (GH)	Yes	[158]
*F. solani*	*CEF559*	75–80%	60% (GH), 30.1–56.3% (field)	No	[159]
*Gibellulopsis nigrescens*	CVn-WHg	Not tested	40.6–95% (GH)	No	[149]
*Leptosphaeria* sp.	CEF-714	Not tested	47.6–58.2% (GH), 50.2–69.2% (field)	No	[157]
*Nectria haematococca*	Bx247	Not tested	69.8% (GH)	Yes	[158]
*Phomopsis* sp.	By231	Not tested	63.4% (GH)	Yes	[158]
*Talaromyces flavus*	CEF-642	Not tested	29.5–26% (GH), 23.2–45.7% (field)	No	[157]
*Trichoderma kogingiopsis*	T2	70.6%	70% (GH)	Not tested	[160]
*T. virens*	G4, G6	Not tested	16–18% (GH)	Yes	[161]

## Data Availability

The original contributions presented in this study are included in the article. Further inquiries can be directed to the corresponding author.
